# Bladder carcinomas and normal urothelium universally express gp200-MR6, a molecule functionally associated with the interleukin 4 receptor (CD 124).

**DOI:** 10.1038/bjc.1996.77

**Published:** 1996-02

**Authors:** M. F. Tungekar, K. C. Gatter, M. A. Ritter

**Affiliations:** Division of Histopathology, United Medical and Dental Schools of Guy's and St Thomas' Hospitals Trust, London, UK.

## Abstract

**Images:**


British Journal of Cancer (1996) 73, 429-432
© 1996 Stockton Press All rights reserved 0007-0920/96 $12.00

### Bladder carcinomas and normal urothelium universally express gp200-MR6, a molecule functionally associated with the interleukin 4 receptor (CD 124)

MF Tungekar[1], KC Gatter[2] and MA Ritter[3]

[1]*Division of Histopathology, United Medical and Dental Schools of Guy's and St Thomas' Hospitals Trust, Lambeth Palace Road, London SE1 7EH, UK;* [2]*Nuffield Department of Pathology, John Radcliffe Hospital, Headington, Oxford OX3 9DU, UK;* [3]*Department of Immunology, Royal Postgraduate Medical School, Hammersmith Hospital, Du Cane Road, London W12 ONN, UK.*

**Summary** Monoclonal antibody MR6 detects gp200-MR6, a molecule functionally associated with the interleukin 4 (IL-4) receptor. Positive immunolabelling with MAb MR6 was obtained in 28/28 transitional cell carcinomas of the bladder, representing a range of different grades and stages of disease, as well as in all control non-neoplastic urothelia. The expression of mutant p53 protein and epidermal growth factor receptor was detected in 14/28 and 20/28 cases respectively. Proliferation indices, determined by Ki67 labelling, ranged from 5% to 95% among these tumours. The universal expression of gp200-MR6 in neoplastic and non-neoplastic urothelium has important implications for the possible use of IL-4 in tumour therapy and suggests that IL-4 may play a role in differentiation and homeostasis of urothelium and other mucosal epithelia.

**Keywords:** interleukin 4; interleukin 4 receptor; adoptive immunotherapy; tumour targeting; bladder neoplasm; mucosal immunity

Adoptive immunotherapy of tumours using cytokines to promote an anti-tumour response mounted by lymphocytes, macrophages and killer cells has attracted a great deal of attention in recent years (Colombo and Forni, 1994). Much of this effort has been directed at interleukin 2 (IL-2). When used alone for this purpose it is effective for only a few tumour types and carries risks of severe toxic reactions (Bukowski *et al.*, 1993). Other members of the interleukin family also hold much promise, given the known interactions amongst the various cytokines (Jansen *et al.*, 1990; Colombo and Forni, 1994). Interleukin 4 (IL-4), the product of a subset of T lymphocytes (Th2), was initially believed to be a B-cell growth factor but is now known to have potent regulatory effects on haemopoietic cells of various lineages and regulates many of the effects of IL-2 (Jansen *et al.*, 1990; Paul, 1991; Isakson, 1992; Paul and Seder, 1994). Therefore, there is much enthusiasm about potential uses of IL-4 in this field (Lotze *et al.*, 1990; Costello *et al.*, 1993; Tepper *et al.*, 1993), especially since it potentiates the anti-proliferative effects of tumour necrosis factor $\alpha$ and interferon $\gamma$ on neoplastic cell lines, may itself have such effects and is tumoricidal in murine models (Totpal *et al.*, 1991; Redmond *et al.*, 1992; Topp *et al.*, 1993).

IL-4 mediates its biological effects by binding to a specific receptor present on the surface of target cells, which is a member of the haematopoietin receptor family (Keegan and Pierce, 1994). The IL-4 receptor (IL-4R), which comprises the IL-4 binding component (CD124) together with the common $\gamma$-chain ($\gamma$c) is expressed by B and T lymphocytes, macrophages and mast cells (Jansen *et al.*, 1990; Russel *et al.*, 1993). Increasingly however its presence is being reported on non-haemopoietic cells such as cell lines derived from melanoma and various epithelial tumours (Morisaki *et al.*, 1992; Toi *et al.*, 1992; Obiri *et al.*, 1993, 1994).

The monoclonal antibody (MAb) MR6 was raised against human thymic cortical epithelial cells but was also found to show weaker reactions with lymphocytes, dendritic cells, macrophages and some non-thymic epithelia (DeMaagd *et al.*, 1985; von Gaudecker *et al.*, 1995). Subsequent investigations showed that MAb MR6 detects a 200 kDa glycoprotein (gp200-MR6) and that addition of the MAb to *in vitro* assays blocks IL-4-induced proliferation of cloned T cells and the IL-4-dependent switch to IgE in allergen-stimulated B cells, and may inhibit the development and expansion of IL4-secreting 'Th2' T helper cells; these data indicate that gp200-MR6 is functionally associated with the IL-4R, although MAb MR6 does not interfere with the binding of IL-4 to the CD124 ligand-binding chain of the receptor (Larché *et al.*, 1988; Mat *et al.*, 1990; Imami *et al.*, 1994).

We have previously used MAb MR6 to demonstrate the presence of gp200-MR6 in surgically resected breast, pulmonary and colorectal carcinomas (Mat *et al.*, 1990; Tungekar *et al.*, 1991; Kaklamanis *et al.*, 1992). However, not all tumours were gp200-MR6 positive, and a subsequent detailed analysis of breast tumours showed that loss of this molecule was associated with higher grade tumours (Mat *et al.*, 1993). These observations not only suggest that gp200-MR6 expression may be useful in prognostic evaluation, but also raise the possibility of direct effects of IL-4 on epithelial tumours in addition to its recognised immunoregulatory influences on the cells infiltrating the tumour stroma.

Intravesical immunotherapy with BCG is a well-established method of treatment for superficial transitional cell carcinomas of the bladder (Ozen, 1993). For advanced forms of these tumours arterial infusion of recombinant interleukin 2 (rIL-2) with lymphokine-activated killer cells has been tried with mixed results (Hermann *et al.*, 1992). The application of IL-4 in this area is likely to be explored in future in view of the synergistic effects it shares with other cytokines. For this reason a study of IL-4R and gp200-MR6 expression in transitional cell carcinomas of bladder is particularly relevant.

We have therefore used MAb MR6 to investigate the expression of gp200-MR6 in a series of 28 resected urothelial bladder carcinomas of various grades and stages. Our data reveal that it is universally expressed by these tumours and by non-neoplastic urothelium. In contrast, the tumours were heterogeneous in their expression of epidermal growth factor receptors (EGFRs) and mutant p53, and exhibited a broad range of proliferative grades as determined by Ki67 staining.

Correspondence: MF Tungekar
Received 24 October 1994; revised 16 August 1995; accepted 11 September 1995

### Materials and methods

Blocks from 28 consecutive surgically excised bladder tumours were snap frozen and stored in liquid nitrogen. The tumour types were classified according to the World Health Organization Classification (Mostofi *et al.*, 1973) and staged according to UICC guide lines (International Union Against Cancer, 1987). The tumours were all transitional cell carcinomas; of these, 12 were grade 2, and 16 grade 3. Depths of invasion and grades are summarised in Table I. Twelve specimens of non-neoplastic urothelium were available as controls: five of these were excised with tumours and the rest obtained at cystoscopic examination that turned out to be substantially normal.

The primary MAb antibodies used are indicated in Table II. All are murine IgG1 reagents except EGFR1 which belongs to the IgG 2 class. Staining with Ki67 was carried out to study the proliferation rates and to assess the general state of preservation of antigens since the Ki67 antigen is highly sensitive to degradation.

Immunohistochemical staining was performed using the alkaline phosphatase–anti-alkaline phosphatase procedure as described previously (Cordell *et al.*, 1984). No staining was observed when the primary antibody was omitted or when it was replaced by an irrelevant isotype-matched monoclonal antibody (Figure 1).

### Results

A total of 28/28 transitional cell carcinomas of the bladder expressed the gp200-MR6. Nearly all the cells within these tumours stained with the MAb MR6, although the intensity of the staining varied from moderate to strong with different areas of each tumour (Figure 2). The staining was mostly concentrated on the cell surfaces and was more intense at the periphery of tumour masses than at their centre. However the intensity of immunoreactivity showed no significant relationship to the grades or stages of the tumours (Figure 3). The results of immunostaining for EGFR and p53 positivity are summarised in Table III. EGFR and p53 positivity were seen in 20/28 and 14/28 cases respectively. The Ki67 positivity rate of the individual tumours ranged from 5% to 95%.

In all the control specimens, non-neoplastic urothelium showed labelling with MAb MR6, with more intense staining of the basal layers and hyperplastic von Brunn's nests (Figure 4). The lymphoid cells infiltrating the tumour stroma were strongly labelled with MAB MR6 (Figure 3).

**Table I** Stages and grades of tumours

| *Depth of invasion* | *Grade* [a] | *Stage* [b] |
|---|---|---|
| Non-invasive, papillary ($n$=11) | G2 ($n$=11) | pTa ($n$=11) |
| Tunica propria ($n$=9) | G2 ($n$=5), G3 ($n$=4) | pT1 ($n$=9) |
| Muscularis propria ($n$=8) | G3 ($n$=8) | pT2 ($n$=8) |

[a]According to WHO Classification of bladder tumours (Mostofi *et al.*, 1973). [b]According to UICC staging protocol for bladder tumours (International Union Against Cancer, 1987).

**Table II** The antibody panel used

| *Antibody* | *Specificity* | *Source* |
|---|---|---|
| MR6 | gp200-MR6 | MA Ritter |
| EGFR1 | Epidermal growth factor receptor | Dako |
| Ki67 | Proliferation-associated antigen | Dako |
| NCL-p53-1801 | Amino acids 32–79 of p53 protein | Novocastra |

### Discussion

The importance of IL-4 as a potent regulatory molecule for haemopoietic cells and its modulatory influence over the effects of other cytokines are well established (Jansen *et al.*, 1990). Its use in the adoptive immunotherapy of tumours is therefore being advocated (Lotze *et al.*, 1990; Costello *et al.*,

**Figure 1** Negative control for alkaline phosphatase immunostaining. No labelling was seen when the primary antibody was replaced by an isotype matched control. (Light haematoxylin nuclear counterstain). Magnification, ×120.

**Figure 2** A grade II, stage pT1 transitional cell carcinoma stained with MAb MR6 shows varying degrees of labelling of tumour cells (t), however the staining is more intense at the cell surfaces as well as along the periphery of tumour cell masses at their interface with stroma (s). Alkaline phosphatase immunostaining. Magnification, ×120.

**Figure 3** A transitional cell carcinoma of similar grade and stage to the tumour in Figure 2 shows weaker labelling with MAb MR6. Note the intense labelling of an aggregate of stromal lymphoid cells (arrow). Alkaline phosphatase immunostaining. Magnification, ×120.

**Figure 4** The non-neoplastic control specimen shows labelling of the surface urothelium (u) as well as of von Brunn's nests (large arrow heads) with MAb MR6. Occasional lymphoid cells in the stroma (small arrow heads) are also labelled. Alkaline phosphatase immunostaining. Magnification, ×120.

**Table III** Pattern of EGFR and p53 staining in 28 cases

| *Antigen* | *EGFR-positive* | *EGFR-negative* |
|---|---|---|
| p53 positive | 9 | 5 |
| p53 negative | 11 | 3 |

1993; Tepper *et al.*, 1993). Topical immunotherapy with BCG is of proven value in superficial but not deeply invasive or metastatic urothelial carcinomas of the bladder (Ozen, 1993). Treatment of advanced carcinomas of these types with infusions of rIL-2 with lymphokine-activated killer (LAK) cells has not had much success and also has toxic side-effects (Hermann *et al.*, 1992; Ozen, 1993). Recombinant IL-4 (rIL-4) enhances the proliferation of tumour infiltrating lymphocytes (TILs) and macrophages induced by IL-2 in bladder carcinomas (Kawakami *et al.*, 1993), and therefore appears to be a logical addition to IL-2 in treatment of these tumours.

Our demonstration that gp200-MR6, which is functionally associated with the IL-4R, is expressed on neoplastic urothelium and other epithelia raises important questions about such possible therapeutic applications of IL-4 (Larché *et al.*, 1988; Mat *et al.*, 1990; Tungekar *et al.*, 1991; Kaklamanis *et al.*, 1992; Mat *et al.*, 1993; Imami *et al.*, 1994). The IL-4R expressed by carcinomas are likely to be functionally active since *in vitro* studies have shown their ability to bind IL-4 with subsequent internalisation of IL-4–IL-4R complex (Morisaki *et al.*, 1992; Toi *et al.*, 1992; Obiri *et al.*, 1993). Thus IL-4, besides modifying the activities of host cells infiltrating the tumours, may directly influence growth of tumour cells. *In vitro* studies have shown that IL-4 alone may induce either inhibition or promotion of growth in cell lines derived from various tumours (Totpal *et al.*, 1991; Morisaki *et al.*, 1992, 1994; Toi *et al.*, 1992; Obiri *et al.*, 1993, 1994). Clinical studies of certain subsets of breast cancers suggest that cytokines released from host cells infiltrating tumour stroma may act in a paracrine fashion to promote growth of neoplastic cells and that cytotoxic therapy is beneficial because of its immunosuppressive effects (Stewart and Tsai, 1993). The expression of gp200-MR6 by non-neoplastic urothelium as well as all the urothelial carcinomas may point to a fundamental physiological role for IL-4 in homeostasis of this tissue that is retained in its neoplastic transformation.

The finding that all bladder carcinomas express gp200-MR6 is in contrast to the selective pattern of expression of this molecule in tumours of lung, breast, colon and rectum (Mat *et al.*, 1990, 1993; Tungekar *et al.*, 1991; Kaklamanis *et al.*, 1992). Thus approximately 35% of all non-small-cell carcinomas of the lung and 30% of breast carcinomas were positive for gp200-MR6, whereas all colorectal adenomas and 90% of carcinomas expressed the antigen (Mat *et al.*, 1990, 1993; Tungekar *et al.*, 1991; Kaklamanis *et al.*, 1992). Moreover, loss of gp200-MR6 in carcinoma of the breast has been shown to be associated with increased malignancy (Mat *et al.*, 1993). The retention of gp200-MR6 expression by all transitional bladder cell carcinomas may give the impression that the cohort of tumours in the present study was biologically homogeneous. That this is unlikely to be the case is illustrated by the obvious heterogeneity of the tumours in terms of known markers of biological behaviour such as grade, stage, proliferative activity determined by Ki67 labelling, and expression of EGFR and mutant p53. These observations indicate that IL-4 may play a role in maintaining the integrity of transitional epithelium even in its neoplastic state and that gp200-MR6 expression is unlikely to be useful in predicting the biological behaviour of bladder tumours. Moreover, while it should be possible to use antibodies such as MR6 with radioisotopic tags for visualisation of primary and metastatic disease in urothelial carcinomas, as previously shown for the lung, the presence of IL-4R on normal epithelia would argue against the use of chimaeric toxin–IL-4 conjugates to target IL-4R positive tumours because of the risks of epithelial damage (Al Jabaari *et al.*, 1989; Lakkis *et al.*, 1991; Debinski *et al.*, 1993).

In conclusion, this study, by demonstrating gp200-MR6 on normal and neoplastic urothelium, has further extended the list of target cells that may be susceptible to the influence of IL-4 and has raised new issues regarding the application of this versatile cytokine in adoptive immunotherapy of tumours. It has also highlighted the possible physiological influence of IL-4 on epithelia and emphasises the importance of studying the expression of receptors in surgical biopsy material in addition to the *in vitro* studies performed on cell lines and in animal models.

**Acknowledgements**
We are grateful to Mr Asit Das and Miss Beryl Evans, United Medical and Dental School (at St Thomas' Hospital), for their help in preparing illustrations and for additional immunohistochemistry respectively.

### References

AL JABAARI B, LADYMAN HM, LARCHE M, SIVOLAPENKO GB, EPENETOS AA AND RITTER MA. (1989). Elevated expression of the interleukin 4 receptor in carcinoma: a target for immunotherapy? *Br. J. Cancer*, **59**, 910–914.

BUKOWSKI, RM, MCLAIN D, OLENCKI T, BUDD GT AND MURTHY SA. (1993). Interleukin-2: use in solid tumors. *Stem Cells (Dayt).*, **11**, 26–32.

COLOMBO MP AND FORNI G. (1994). Cytokine transfer in tumor inhibition and tumor therapy: where are we now? *Immunol. Today*, **15**, 48–51.

CORDELL JL, FALINI B, ERBER W, GHOSH A, ABDULAZIZ Z, MACDONALD S, PULFORD KAS, STEIN H AND MASON DY. (1984). Immunoenzymatic labelling of monoclonal antibodies using immune complexes of alkaline phosphatase and monoclonal anti-alkaline phosphatase (APAAP). *J. Histochem. Cytochem.*, **32**, 219–229.

COSTELLO R, LIPCEY C AND OLIVE D. (1993). Rationale for the utilization of interleukin-4, an immune-recognition induced cytokine, in cancer immunotherapy (review). *Eur. J. Med.*, **2**, 54–57.

DEBINSKI W, PURI RK, KREITMAN RJ AND PASTAN I. (1993). A wide range of human cancers express interleukin 4 (IL4) receptors that can be targeted with chimeric toxin composed of IL-4 and Pseudomonas exotoxin. *J. Biol. Chem.*, **268**, 14065–14070.

DE MAAGD RA, MACKENZIE WA, SCHUURMAN HJ, RITTER MA, PRICE KM, BROEKHUIZEN R AND KATER L. (1985). The human thymus microenvironment; heterogeneity detected by monoclonal antiepithelial antibodies. *Immunology*, **54**, 745–754.

HERMANN GG, GEERTSEN PF, VON DER MAASE H, STEVEN K, ANDERSEN C, HALDT T AND ZEUTHEN J. (1992). Recombinant interleukin-2 and lymphokine-activated killer cell treatment of advanced bladder cancer: clinical results and immunological effects. *Cancer Res.*, **52**, 726–733.

IMAMI N, LARCHE M AND RITTER MA. (1994). Inhibition of alloreactivity by monoclonal antibody MR6: differential effects on IL-2- and IL-4-producing human T-cells. *Int. Immunol.*, **6**, 1575–1584.

INTERNATIONAL UNION AGAINST CANCER. (1987). *TNM Classification of Malignant Tumours*. Springer: Berlin.

ISAKSON, P. (1992). Interleukin 4. *Adv. Neuroimmunol.*, **2**, 55–58.

JANSEN JH, FIBBE WE, WILLEMZE R AND KLUIN-NELEMANS JC. (1990). Interleukin-4. A regulatory protein. *Blut*, **60**, 269–274.

KAKLAMANIS L, GATTER KC, MORTENSEN N AND HARRIS AL. (1992). Interleukin-4 receptor and epidermal growth factor receptor expression in colorectal cancer. *Br. J. Cancer*, **66**, 712–716.

KAWAKAMI Y, HAAS GP AND LOTZE MT. (1993). Expression of tumor-infiltrating lymphocytes from human tumors using the T-cell growth factors interleukin-2 and interleukin-4. *J. Immunother.*, **14**, 336–347.

KEEGAN AD AND PIERCE JH. (1994). The interleukin-4 receptor: signal transduction by a hematopoietin receptor (review). *J. Leukoc. Biol.*, **55**, 272–279.

LAKKIS F, STEELE A, PACHECO-SILVA A, RUBIN-KELLEY V, STROM TB AND MURPHY JR. (1991). Interleukin 4 receptor targeted cytotoxicity: genetic construction and *in vivo* immunosuppressive activity of a diphtheria toxin-related murine interleukin 4 fusion protein. *Eur. J. Immunol.*, **21**, 2253–2258.

LARCHÉ M, LAMB JR, O'HEHIR RE, IMAMI-SHITA N, ZANDERS ED, QUINT DE, MOQBEL R AND RITTER MA. (1988). Functional evidence for a monoclonal antibody that binds to the human interleukin-4 receptor. *Immunology*, **65**, 617–622.

LOTZE MT, CUSTER MC, BOLTON ES, WIEBKE EA, KAWAKAMI Y AND ROSENBERG SA. (1990). Mechanisms of immunological antitumor therapy: lessons from the laboratory and clinical applications (review). *Hum. Immunol.*, **28** (2), 198–207.

MAT I, LARCHE M, MELCHER D AND RITTER MA. (1990). Tumour-associated upregulation of the IL-4 receptor complex. *Br. J. Cancer*, **10** (suppl.) 96–98.

MAT IB, MOORS N, MELCHER D, FOXWELL BMJ AND RITTER MA. (1993). Differential expression of MR6 antigen and c-erbB-2 protein in breast tumours. In *Mutant Oncogenes: Targets of Therapy*. Lemoine N and Epenetos AA (eds) pp. 53–64. Chapman and Hall: London.

MORISAKI T, YUZUKI D, LIN RT, FOSHAG LJ, MORTON DL AND HOON DS. (1992). Interleukin 4 receptor expression and growth inhibition of gastric carcinoma cells by interleukin 4. *Cancer Res.*, **52**, 6059–6065.

MORISAKI T, UCHIYAMA A, YUZUKI D, ESSNER R, MORTON DL AND HOON DS. (1994). Interleukin 4 regulates $G_1$ cell cycle progression in gastric carcinoma cells. *Cancer Res.*, **54**, 1113–1118.

MOSTOFI FK, SOBIN LH AND TORLONI H. (1973). *Histological Typing of Urinary Bladder Tumors*. International Classification of Tumors, No. 10. WHO: Geneva.

OBIRI N, HILLMAN GG, HAAS GP, SUD S AND PURI RK. (1993). Expression of high affinity interleukin-4 receptors on human renal cell carcinoma cells and inhibition of tumor cell growth *in vitro* by interleukin-4. *J. Clin. Invest.*, **91**, 88–93.

OBIRI N., SIEGEL JP, VARRICHIO F AND PURI RK. (1994). Expression of high affinity interleukin-4 receptors on human melanoma, ovarian and breast carcinoma cells. *Clin. Exp. Immunol.*, **95**, 148–155.

OZEN H. (1993). Advances in bladder cancer. *Curr. Opin. Oncol.*, **5**, 574–580.

PAUL WE. (1991). Interleukin 4: a prototypic immunoregulatory lymphokine. *Blood*, **77**, 1859–1870.

PAUL WE AND SEDER RA. (1994). Lymphocyte responses and cytokines. *Cell*, **76**, 241–251.

REDMOND HP, SCHUCHTER L, BARTLETT D, KELLY CJ, SHOU J, LEON P AND DALY JM. (1992). Anti-neoplastic effects of interleukin-4. *J. Surg. Res.*, **52**, 406–411.

RUSSEL SM, KEEGAN AD, HARADA H, NAKAMURA Y, NOGUCHI M, LELAND P, FRIEDMANN MC, MIYAJIMA A, PURI RK, PAUL WE AND LEONARD WJ. (1993). Interleukin-2 receptor $\gamma$ chain: A functional component of the interleukin-4 receptor. *Science*, **262**, 1880–1883.

STEWART TH AND TSAI SC. (1993). The possible role of stromal cell stimulation in worsening the prognosis of a subset of patients with breast cancer. *Clin. Exp. Metastasis*, **11**, 295–305.

TEPPER RI. (1993). The anti-tumour and proinflammatory actions of IL4 (review). *Res. Immunol.*, **144**, 633–637.

TOI M, BICKNELL R AND HARRIS AL. (1992). Inhibition of colon and breast carcinoma cell growth by interleukin-4. *Cancer Res.*, **52**, 275–279.

TOPP MS, KOENIGSMANN M, MIRE-SLUIS A, OBERBERG D, EITELBACH F, VON MARSCHALL Z, NOTTER M, REUFL B, STEIN H, THIEL E AND WOLFGANG EB. (1993). Recombinant human Interleukin 4 inhibits growth of some human lung tumor cell lines *in vitro* and *in vivo*. *Blood*, **82**, 2837–2844.

TOTPAL K AND AGGARWAL BB. (1991). Interleukin 4 potentiates the antiproliferative effects of tumor necrosis factor on various tumor cell lines. *Cancer Res.*, **51**, 4266–4270.

TUNGEKAR MF, TURLEY H, DUNNILL MS, GATTER KC, RITTER MA AND HARRIS AL. (1991). Interleukin 4 receptor expression on human lung tumors and normal lung. *Cancer Res.*, **51**, 261–264.

VON GAUDECKER B, KENDALL MD AND RITTER MA. (1995). Immuno-electron microscopy of the thymic microenvironment. *Microscopy Research and Technique* (in press).


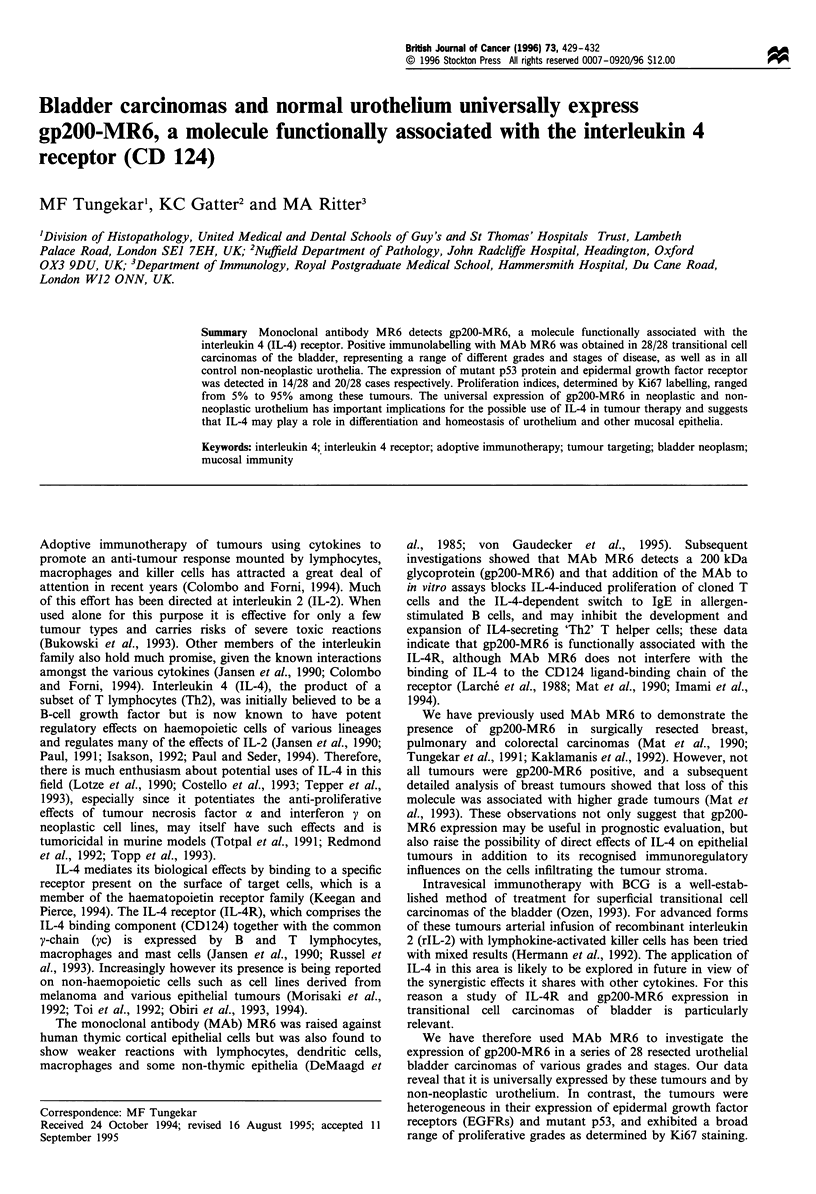

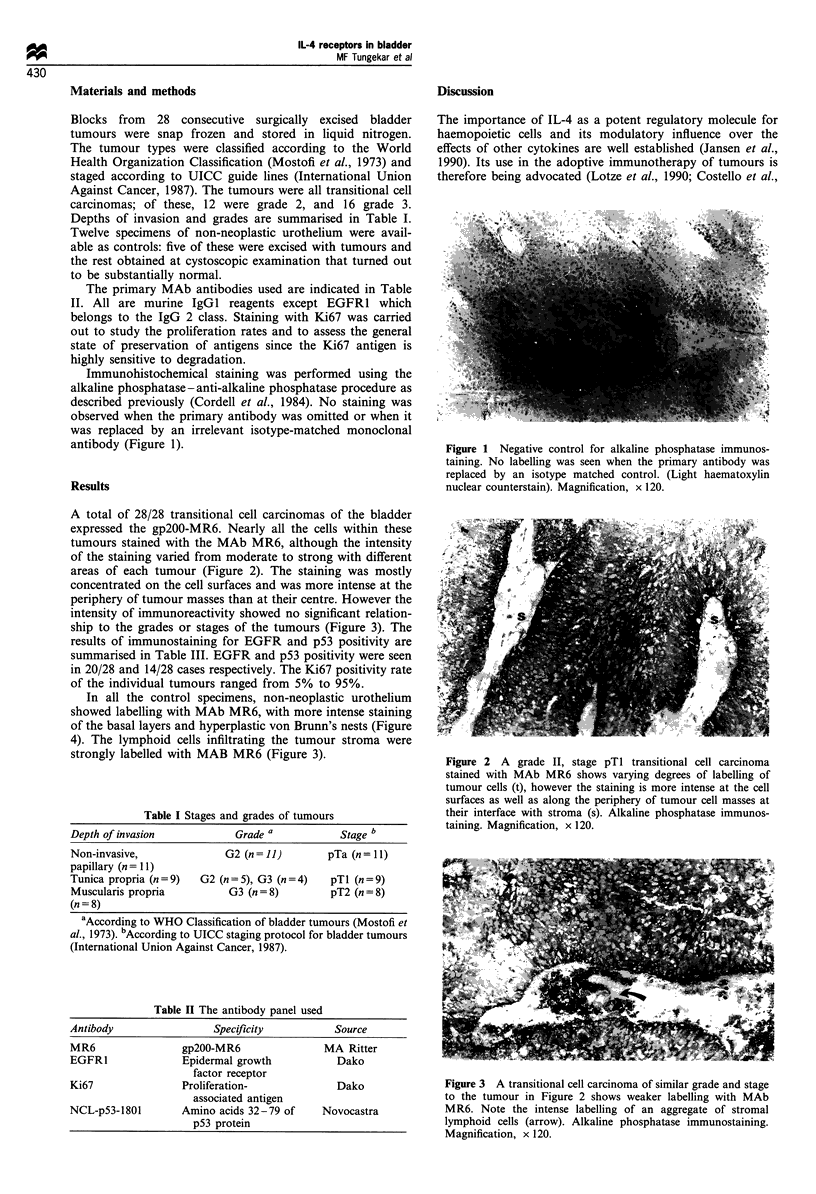

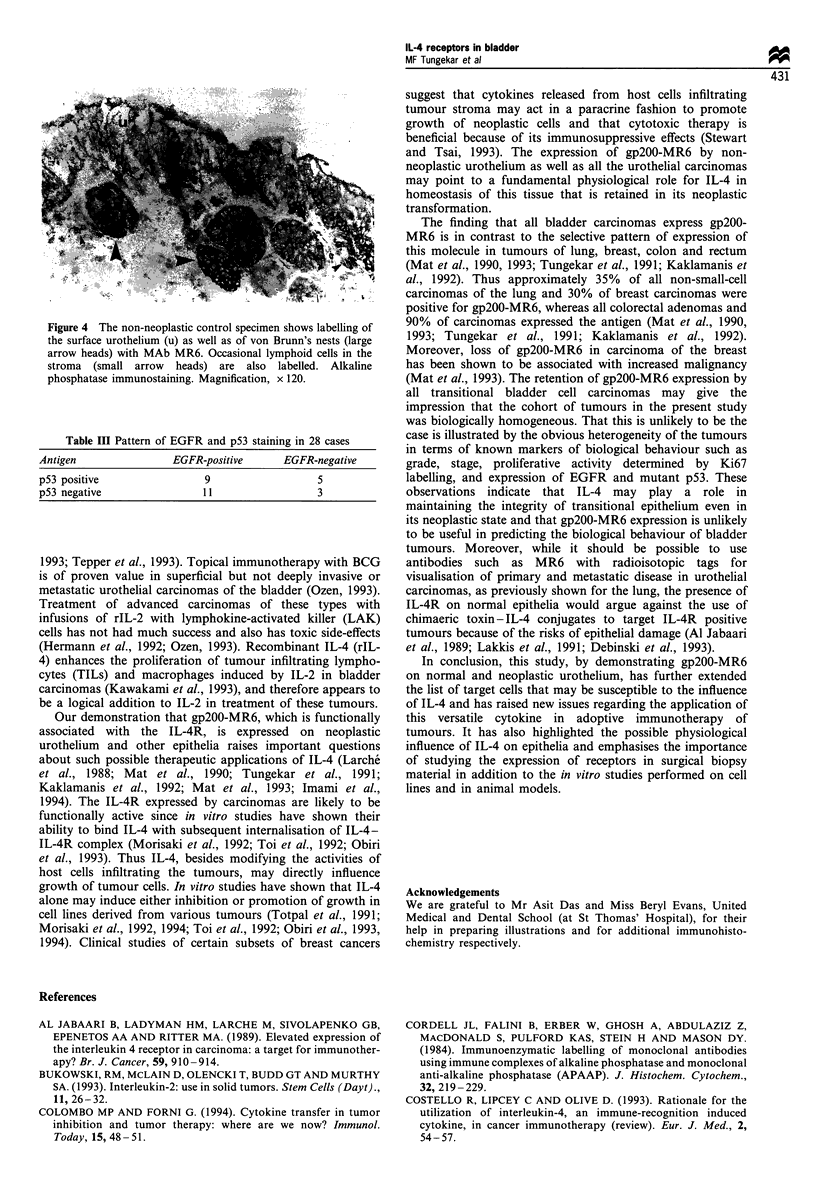

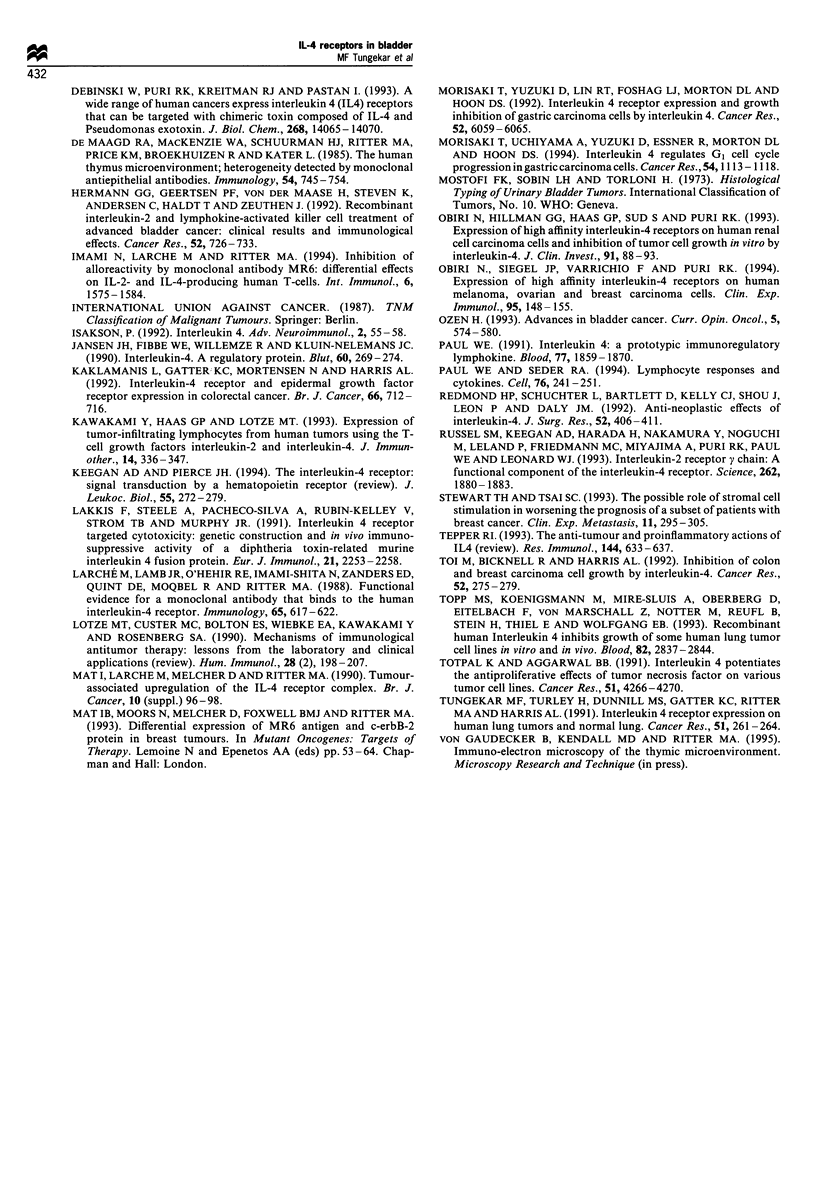


## References

[OCR_00361] Al Jabaari B., Ladyman H. M., Larché M., Sivolapenko G. B., Epenetos A. A., Ritter M. A. (1989). Elevated expression of the interleukin 4 receptor in carcinoma: a target for immunotherapy?. Br J Cancer.

[OCR_00369] Bukowski R. M., McLain D., Olencki T., Budd G. T., Murthy S. A. (1993). Interleukin-2: use in solid tumors.. Stem Cells.

[OCR_00374] Colombo M. P., Forni G. (1994). Cytokine gene transfer in tumor inhibition and tumor therapy: where are we now?. Immunol Today.

[OCR_00379] Cordell J. L., Falini B., Erber W. N., Ghosh A. K., Abdulaziz Z., MacDonald S., Pulford K. A., Stein H., Mason D. Y. (1984). Immunoenzymatic labeling of monoclonal antibodies using immune complexes of alkaline phosphatase and monoclonal anti-alkaline phosphatase (APAAP complexes).. J Histochem Cytochem.

[OCR_00385] Costello R., Lipcey C., Olive D. (1993). Rationale for the utilization of interleukin-4, an immune-recognition induced cytokine, in cancer immunotherapy.. Eur J Med.

[OCR_00397] Debinski W., Puri R. K., Kreitman R. J., Pastan I. (1993). A wide range of human cancers express interleukin 4 (IL4) receptors that can be targeted with chimeric toxin composed of IL4 and Pseudomonas exotoxin.. J Biol Chem.

[OCR_00409] Hermann G. G., Geertsen P. F., von der Maase H., Steven K., Andersen C., Hald T., Zeuthen J. (1992). Recombinant interleukin-2 and lymphokine-activated killer cell treatment of advanced bladder cancer: clinical results and immunological effects.. Cancer Res.

[OCR_00414] Imami N., Larché M., Ritter M. A. (1994). Inhibition of alloreactivity by mAb MR6: differential effects on IL-2- and IL-4- producing human T cells.. Int Immunol.

[OCR_00426] Jansen J. H., Fibbe W. E., Willemze R., Kluin-Nelemans J. C. (1990). Interleukin-4. A regulatory protein.. Blut.

[OCR_00432] Kaklamanis L., Gatter K. C., Mortensen N., Harris A. L. (1992). Interleukin-4 receptor and epidermal growth factor receptor expression in colorectal cancer.. Br J Cancer.

[OCR_00438] Kawakami Y., Haas G. P., Lotze M. T. (1993). Expansion of tumor-infiltrating lymphocytes from human tumors using the T-cell growth factors interleukin-2 and interleukin-4.. J Immunother Emphasis Tumor Immunol.

[OCR_00444] Keegan A. D., Pierce J. H. (1994). The interleukin-4 receptor: signal transduction by a hematopoietin receptor.. J Leukoc Biol.

[OCR_00450] Lakkis F., Steele A., Pacheco-Silva A., Rubin-Kelley V., Strom T. B., Murphy J. R. (1991). Interleukin 4 receptor targeted cytotoxicity: genetic construction and in vivo immunosuppressive activity of a diphtheria toxin-related murine interleukin 4 fusion protein.. Eur J Immunol.

[OCR_00457] Larche M., Lamb J. R., O'Hehir R. E., Imami-Shita N., Zanders E. D., Quint D. E., Moqbel R., Ritter M. A. (1988). Functional evidence for a monoclonal antibody that binds to the human IL-4 receptor.. Immunology.

[OCR_00462] Lotze M. T., Custer M. C., Bolton E. S., Wiebke E. A., Kawakami Y., Rosenberg S. A. (1990). Mechanisms of immunologic antitumor therapy: lessons from the laboratory and clinical applications.. Hum Immunol.

[OCR_00468] Mat I., Larche M., Melcher D., Ritter M. A. (1990). Tumour-associated upregulation of the IL-4 receptor complex.. Br J Cancer Suppl.

[OCR_00487] Morisaki T., Uchiyama A., Yuzuki D., Essner R., Morton D. L., Hoon D. S. (1994). Interleukin 4 regulates G1 cell cycle progression in gastric carcinoma cells.. Cancer Res.

[OCR_00481] Morisaki T., Yuzuki D. H., Lin R. T., Foshag L. J., Morton D. L., Hoon D. S. (1992). Interleukin 4 receptor expression and growth inhibition of gastric carcinoma cells by interleukin 4.. Cancer Res.

[OCR_00526] Noguchi M., Nakamura Y., Russell S. M., Ziegler S. F., Tsang M., Cao X., Leonard W. J. (1993). Interleukin-2 receptor gamma chain: a functional component of the interleukin-7 receptor.. Science.

[OCR_00493] Obiri N. I., Hillman G. G., Haas G. P., Sud S., Puri R. K. (1993). Expression of high affinity interleukin-4 receptors on human renal cell carcinoma cells and inhibition of tumor cell growth in vitro by interleukin-4.. J Clin Invest.

[OCR_00499] Obiri N. I., Siegel J. P., Varricchio F., Puri R. K. (1994). Expression of high-affinity IL-4 receptors on human melanoma, ovarian and breast carcinoma cells.. Clin Exp Immunol.

[OCR_00507] Ozen H. (1993). Advances in bladder cancer.. Curr Opin Oncol.

[OCR_00511] Paul W. E. (1991). Interleukin-4: a prototypic immunoregulatory lymphokine.. Blood.

[OCR_00513] Paul W. E., Seder R. A. (1994). Lymphocyte responses and cytokines.. Cell.

[OCR_00520] Redmond H. P., Schuchter L., Bartlett D., Kelly C. J., Shou J., Leon P., Daly J. M. (1992). Anti-neoplastic effects of interleukin-4.. J Surg Res.

[OCR_00522] Russell S. M., Keegan A. D., Harada N., Nakamura Y., Noguchi M., Leland P., Friedmann M. C., Miyajima A., Puri R. K., Paul W. E. (1993). Interleukin-2 receptor gamma chain: a functional component of the interleukin-4 receptor.. Science.

[OCR_00529] Stewart T. H., Tsai S. C. (1993). The possible role of stromal cell stimulation in worsening the prognosis of a subset of patients with breast cancer.. Clin Exp Metastasis.

[OCR_00534] Tepper R. I. (1993). The anti-tumour and proinflammatory actions of IL4.. Res Immunol.

[OCR_00540] Toi M., Bicknell R., Harris A. L. (1992). Inhibition of colon and breast carcinoma cell growth by interleukin-4.. Cancer Res.

[OCR_00545] Topp M. S., Koenigsmann M., Mire-Sluis A., Oberberg D., Eitelbach F., von Marschall Z., Notter M., Reufi B., Stein H., Thiel E. (1993). Recombinant human interleukin-4 inhibits growth of some human lung tumor cell lines in vitro and in vivo.. Blood.

[OCR_00550] Totpal K., Aggarwal B. B. (1991). Interleukin 4 potentiates the antiproliferative effects of tumor necrosis factor on various tumor cell lines.. Cancer Res.

[OCR_00555] Tungekar M. F., Turley H., Dunnill M. S., Gatter K. C., Ritter M. A., Harris A. L. (1991). Interleukin 4 receptor expression on human lung tumors and normal lung.. Cancer Res.

[OCR_00404] de Maagd R. A., MacKenzie W. A., Schuurman H. J., Ritter M. A., Price K. M., Broekhuizen R., Kater L. (1985). The human thymus microenvironment: heterogeneity detected by monoclonal anti-epithelial cell antibodies.. Immunology.

